# Transcriptomics explores the potential of flavonoid in non-medicinal parts of *Saposhnikovia divaricata* (Turcz.) Schischk

**DOI:** 10.3389/fpls.2023.1067920

**Published:** 2023-02-27

**Authors:** Yuqiu Chen, Tao Zhang, Changbao Chen, Zhefeng Xu, Chunshuo Liu

**Affiliations:** Jilin Ginseng Academy, Changchun University of Chinese Medicine, Changchun, Jilin, China

**Keywords:** *Saposhnikovia divaricata*, transcriptome, flavonoid biosynthesis, network pharmacology, 5-O-methylvesamitol

## Abstract

**Introduction:**

*Saposhnikovia divaricata* is a traditional Chinese medicine in China, which is widely used in clinic. The root of S. divaricata is often used as medicine, but little research has been done on its other tissues.

**Methods:**

In this study, the contents of root and leaf of S. divaricata were determined by HPLC, the differentially expressed genes were screened by transcriptome sequencing at molecular level, and then verified by network pharmacology.

**Results:**

The results showed that the content of 4’-O-β-D-glucosyl-5-O-methylvisamminol in the leaves was significantly higher than that in the roots, which was about 3 times higher than that in the roots. In addition, 10 differentially expressed key enzyme genes were screened in plant hormone signal transduction, phenylpropanoid and flavonoid biosynthetic pathways. C4H and CYP98A were up-regulated in root, while F3H was down-regulated in root. They can be used as important candidate genes for the mechanism of quality difference of S. divaricata. Finally, network pharmacological validation showed that 5-O-methylvesamitol plays an important role in the treatment of ulcerative colitis.

**Discussion:**

These findings not only provide insight into flavonoid biosynthesis in S. divaricata associated molecular regulation, but also provide a theoretical basis for the development and utilization of *S. divaricata*.

## 1 Introduction

Traditional Chinese medicine (TCM) resources are the premise and foundation for the sustainable development of Chinese medicine. With the depletion of resources and the deterioration of ecology, the study on the comprehensive utilization of non-traditional medicinal parts of common medicinal materials has attracted more and more attention. For example, ginseng is commonly used for its roots but studies have shown that the stems and leaves of ginseng also have good antioxidant activity (Chen et al., 2020). It is of great significance for the development and utilization of traditional Chinese medicine resources to develop the applied parts of medicinal materials from the angle of material basis or pharmacodynamics (Zhu et al., 2015).

*Saposhnikovia divaricata* (S. *divaricata*) is derived from the dried roots of *Saposhnikovia divaricata* (Turcz.) Schischk and used as a Chinese herbal medicine for treating respiratory, immune, and nervous system diseases (Zhang T. et al., 2021; Chen et al., 2022). It is mainly produced in Jilin, Liaoning and Heilongjiang provinces in China. The main chemical components of *S. divaricata* are chromogenin, coumarin, polysaccharide, volatile oil and so on (Kong et al., 2013; Chun et al., 2016). Among them, chromogenic ketones are considered to be the most important active components in *S. divaricata*. Chromones, a subclass of flavonoids, are a class of oxygen-containing heterocyclic compounds having a benzo-cyclized γ-pyrone ring. They are the primary active components of *S. divaricata*, and research on these compounds has been well studied than that on other components. Studies have shown that the active components in prim-O-glucosylcimifugin and 4’-*O*-β-D-glucosyl-5-*O*-methylvisamminol can not only effectively inhibit a variety of stimuli caused by pain in mice, but also can effectively reduce the xylene-induced ear swelling in mice (Zhou et al., 2017; Fu et al., 2020). Cimifugin has good antipyretic, analgesic and anti-inflammatory effects (Jia et al., 2016). Sec-*O*-glucosylhamaudol can inhibit the release of NO and IL-6 induced by LPS, and has anti-inflammatory activity (Kim et al., 2017).

Flavonoid is an important secondary metabolite involved in plant development and environmental responses. The flavonoid can enhance plant adaptability to a variety of terrestrial environments and participate in plant ecological defense, such as promoting plant growth, disease resistance, and preventing the invasion of harmful microorganisms. Therefore, flavonoid may be secondary metabolites in response to the environment (Stafford, 1991; Agati et al., 2012; Falcone Ferreyra et al., 2012). Flavonoid is polyphenol compound with many valuable biological functions, including anti-tumor, anti-oxidation, anti-inflammation and so on (Zhang et al., 2017; Liu et al., 2021; Zhang S. et al., 2021). Modern pharmacological studies show that chromogen ketone is the most important chemical component of *S. divaricata*, and chromogen ketones are mainly synthesized by flavonoid biosynthesis pathway (Tai and Cheung, 2007; Kreiner et al., 2017). It has been shown that phenylalanine and tyrosine are precursors of phenylpropane biosynthesis, which is then reacted with L-phenylalanin ammo-nialyase(PAL) to produce cinnamic acid and *p*-coumaric acid. *P*-coumaric acid generates *p*-coumaroyl-CoA under the action of 4-coumarate coenzyme A ligase(4CL), and then transferred to flavonoid biosynthetic pathway by Chalcone synthase(CHS), and Chromone was produced by a series of reactions (Zhang et al., 2017). Among them, CHS is a member of polyketone synthase III (PKSIII) family and a characteristic enzyme in the flavonoid biosynthesis pathway (Nakatsuka et al., 2008). Some studies have shown that CHS is an important regulatory point in the synthesis of chloropropanone, which is the rate-limiting enzyme in the synthesis of chloropropanone (Qi et al., 2017; Yang et al., 2019). In addition, plant hormones play an important role in all stages of plant growth and environmental response. Studies have shown that they not only affect plant growth and development, but also regulate plant secondary metabolism and affect the synthesis of flavonoids, flavonols, anthocyanins and other flavonoid compounds in plants (Rui et al., 2020; Yu et al., 2021). For example, it has been found that indole-3-acetic acid(IAA) mainly delays the biosynthesis and accumulation of endogenous abscisic acid in fruits, inhibits the expression of genes related to flavonoid biosynthesis, and reduces the content of flavonoids such as anthocyanins in fruits, thus delaying the ripening of the fruit (Jeong et al., 2004).Therefore, these key differential genes in the synthetic pathway are of great significance for the study of the synthesis of secondary metabolites.

Transcriptome is a collection of all mRNAs transcribed by a specific tissue or cell in a certain developmental stage or functional state. It can study gene function and gene structure at the overall level, and reveal specific biological processes and molecular mechanisms in the occurrence of diseases (Wang et al., 2014). In recent years, the technology of transcriptome sequencing has been widely used in the study of traditional Chinese medicine, and has been applied to Acanthopanax senticosus (Li et al., 2021), atractylodes macrocephala (Ruan et al., 2021), Angelica (Zhao et al., 2021) and other Chinese medicinal materials. These studies are helpful to reveal the formation mechanism of effective active components of traditional Chinese medicine. At the same time, network pharmacology is also rising in the world. It is based on the theories of system biology, genomics and proteomics, and reveals the network relationship of drug-gene-target-disease interaction. Then the network relationship was used to predict the mechanism of drug action to evaluate the drug efficacy and adverse reactions (Hong et al., 2017; Wang et al., 2017).

In recent years, the research hotspots of *S. divaricata* mainly focus on its pharmacological effects, processing technology and quality evaluation of its origin. However, little research has been done on the synthetic mechanism of flavonoid accumulation, which plays an important role in *S. divaricata*, and the development and utilization of non-medicinal components (Ma et al., 2018; Ding et al., 2020; Sun et al., 2022). Therefore, we studied the roots and leaves of S. *divaricata* in order to explore the possibility that its leaves could be used medicinally. prim-*O*-glucosylcimifugin, 4’-*O*-β-D-glucosyl-5-*O*-methylvisamminol, cimifugin, and sec-*O*-glucosylhamaudol play important pharmacological roles in *S. divaricata* with high content(Wang et al., 1999; Crown et al., 2006; Chen et al., 2013; Li et al., 2015). Therefore, the contents of these four chromogenic ketones in the roots and leaves of *S. divaricata* were determined by HPLC, transcriptome sequencing was used to screen for key differentially expressed genes present between roots and leaves of *S. divaricata*, and to predict the molecular mechanisms and biosynthetic pathways of component differences between roots and leaves of *S. divaricata*. In addition, network pharmacology was used to verify some of the compounds in S. divaricata that play a major role in the treatment of specific diseases, such as ulcerative colitis. The importance of 4’-*O*-β-D-glucosyl-5-*O*-methylvisamminol was verified from the side, which showed that the leaves of *S. divaricata* with high content of 4’-*O*-β-D-glucosyl-5-*O*-methylvisamminol also had broad prospects and development value. This study not only provides a theoretical basis for exploring the biosynthesis mechanism of active components in *S. divaricata*, but also provides a new idea for the utilization and development of Chinese medicinal resources.

## 2 Materials and methods

### 2.1 Plant materials

**Figure 1:**
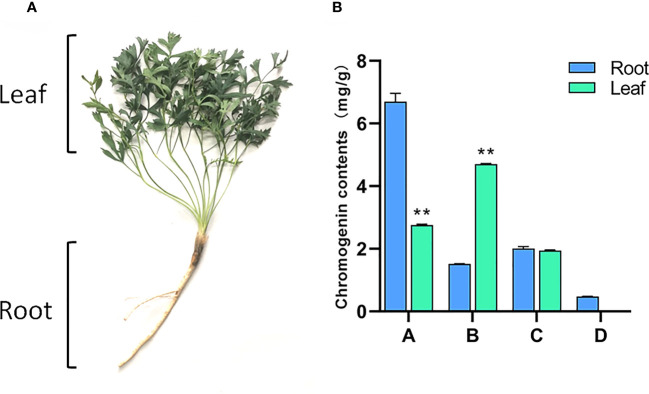
Root and leaf of S. *divaricate*. **(A)** Morphological difference of root and leaf of S. *divaricate*;**(B)** Chromogenin contents in root and leaves. **(**Note: A; prim-*O*-glucosylcimifugin; B; 4’-*O*-β-D-glucosyl-5-*O*-methylvisamminol; C; cimifugin; D; sec-*O*-glucosylhamaudol. Compared with the root, **: Indicates extremely significant difference (P<0.01).

Samples were collected from three-year-old *S. divaricata*, cultivated on the herb garden of Changchun University of Chinese Medicine, Nanguan District, Changchun City, Jilin Province, China(43°49’N, 125°25’E) at the end of August 2020. The collected samples were divided into two tissue sites, root and leaf, as shown in **Figure 1A**. Three biological replicates were set for each tissue site. Two samples were collected and placed in self-sealing bags. One was used for HPLC content determination and the other was frozen in liquid nitrogen and sent to Hangzhou Lianchuan Biology Co., Ltd. for RNA extraction and transcriptome sequencing analysis.

### 2.2 Determination of the chromone content

After drying the samples, these were ground into a powder. The ground powder was filtered through a 60-mesh sieve and then weighed in three replicates, each weighing 1.0 g. Chromogen ketones were extracted ultrasonically (extraction conditions: ultrasonic frequency, 40 kHz; extraction temperature, 30°C;extraction time, 30 min;liquidratio,1:15), extracted three times with methanol, filtered in a funnel, and then the filtered liquids were combined. After the filtrate was evaporated in an evaporating dish and transferred to a 5 ml volumetric flask, the volume was adjusted to 5 ml and the solution was filtered using a 0.22 μm filter. prim-*O*-glucosylcimifugin (80681-45-4), cimifugin(37921-38-3), 4’-*O*-β-D-glucosyl-5-*O*-methylvisamminol(84272-85-5), and sec-*O*-glucosylhamaudol(80681-44-3) were all supplied by Shanghai YUANYE Biotechnology Co., Ltd. The purity of each ingredient was greater than 98% as determined by HPLC. Appropriate amounts of prim-*O*-glucosylcimifugin, cimifugin, 4’-*O*-β-D-glucosyl-5-*O*-methylvisamminol, and sec-*O*-glucosylhamaudol were precisely weighed, and then methanol was added. High-performance liquid chromatography was performed using an Agilent 1,200 series high-performance liquid chromatography system (Agilent, Palo Alto, CA, United States), equipped with an autosampler and a UV detector with a C18 column (4.6 mm × 250 mm, 5 μm; Agilent). Gradient elution was performed using solvent A (100% methanol) and solvent B (100% water) at 30°C, according to the following gradient program: 0–20 min, 30% A; 20–25 min, 45% A; 25–50 min, 60% A; 50–55 min, 90% A; and 55–60 min, 100% A. The flow rate was maintained at 1.0 ml/min, the sample injection volume was 10 μl, and UV absorption was measured at 254 nm. A mixed solution containing all reference substances was prepared and diluted with methanol to obtain 4 reference substance solutions of different concentrations. Mixed reference solutions of different concentrations were used to draw the standard curve. As shown in **Supplementary Table S1**, a good calibration curve was obtained for the four compounds, with a high correlation coefficient (R^2^ > 0.997) and a good linearity over a wide range. The HPLC chromatogram of the S. divaricata reference solution is shown in **Supplementary Figure S1A**, and the HPLC chromatogram of the sample solution is shown in **Supplementary Figure S1B**.

### 23 Transcriptome sequencing and analysis

#### 23.1 RNA extraction, library construction and transcriptome sequencing analysis

Total RNA was extracted using Trizol reagent (Invitrogen, CA, USA) following the manufacturer’s procedure. The total RNA quantity and purity were analysis of Bioanalyzer 2100 and RNA 1000 Nano LabChip Kit (Agilent, CA, USA) with RIN number >7.0. Poly (A) RNA is purified from total RNA (5ug) using poly-T oligo-attached magnetic beads using two rounds of purification. Following purification, the mRNA is fragmented into smallpieces using divalent cations under elevated temperature. Then the cleaved RNA fragments werereverse-transcribed to create the final cDNA library in accordance with the protocol for the mRNASeq sample preparation kit (Illumina, San Diego, USA), the average insert size for the paired-endlibraries was 300 bp ( ± 50 bp). And then we performed the paired-end sequencing on an Illumina Novaseq™ 6000 at the (LC Sceiences,USA) following the vendor’s recommended protocol.

#### 2.3.2 *De novo* assembly, unigene annotation and functional classification

Firstly, Cutadapt and perl scripts in house were used to remove the reads that contained adaptor contamination, low quality bases and undetermined bases. Then sequence quality was verified using FastQC (http://www.bioinformatics.babraham.ac.uk/projects/fastqc/). including the Q20, Q30 and GC-content of the clean data. All downstream analyses were based on clean data of high quality. *De novo* assembly of the transcriptome was performed with Trinity 2.4.0 (Grabherr et al., 2011). Trinity groups transcripts into clusters based on shared sequence content. Such a transcript cluster is very loosely referred to as a ‘gene’. The longest transcript in the cluster was chosen as the ‘gene’ sequence (aka Unigene).

All assembled Unigenes were aligned against the non-redundant (Nr) protein database (http://www.ncbi.nlm.nih.gov/),Gene ontology (GO)(http://www.geneontology.org), SwissProt(http://www.expasy.ch/sprot/), Kyoto Encyclopedia of Genes and Genomes (KEGG)(http://www.genome.jp/kegg/) and eggNOG(http://eggnogdb.embl.de/) databases using DIAMOND(Buchfink et al., 2015) with a threshold of Evalue<0.00001.

#### 2.3.3 Differentially expressed Unigene analysis

Salmon (Patro et al., 2017) was used to perform expression level for Unigenes by calculating TPM (Mortazavi et al., 2008). The differentially expressed Unigenes were selected with log2 (fold change) >1 or log2 (fold change) <-1 and with statistical significance (p value < 0.05) by R package edger (Robinson et al., 2010).

#### 2.3.4 Quantitative reverse transcription-PCR

The instructions of the TaKaRa MiniBEST Universal RNA Extraction Kit were followed for total RNA extraction (TaKaRa, Kusatsu, Shiga, Japan), and the PrimeScriptTM RT Master Mix Kit (TaKaRa) was used for reverse transcription. RT-qPCR was performed on a 96-well plate using an Agilent Technologies Stratagene Mx3000P thermal cycler and a SYBR Green-based PCR kit. Each reaction involved 1 μl of cDNA template (1 mg/ml), 10 μl of SYBR Green Mix (TaKaRa), 1 μl of forward primer (1 mM), 1 μl of reverse primer (1 mM), and 7 μl of ddH2O. The thermal conditions were as follows: 95°C for 3 min, followed by 40 cycles at 95°C for 5 s, 55°C for 32 s, and 72°C for 20 s. The melting curve was obtained by gradually increasing the temperature from 55°C to 95°C at a heating rate of 0.1°C/s. RT-qPCR analysis was performed using three biological replicates. To validate the reliability of transcriptome data, 10 differentially expressed genes with high or low expression levels were randomly selected for RT-qPCR validation. *GAPDH* (glyceraldehyde-3-phosphate dehydrogenase) was used as a housekeeping gene to estimate the relative gene expression using the 2−ΔΔCt method. The primer sequences used in this study are shown in **Supplementary Table S2**.

### 24 Network pharmacology

#### 2.4.1 Software and databases used in network pharmacology

In the section on network pharmacology, the treatment of ulcerative colitis with *S. divaricata* is taken as an example. The TCMSP (https://tcmspw.com/) database was used to collect the chemical composition of *S. divaricata*, and the Gene Cards (https://www.Genecards.org/) database was used to collect ulcerative colitis genes, the Uniport (http://www.uniprot.org/) database was used to standardize proteins.

#### 2.4.2 Acquisition of active components and disease targets of *Saposhnikovia divaricata*

All the active ingredients of *S. divaricata* were collected through the TCMSP database, and the two indicators of oral bioavailability (OB) ≥ 30% and drug-like index (DL) ≥ 0.18 were used as screening thresholds. Finally, we combined the literature to determine the final drug active ingredient. The potential target proteins corresponding to the active components were collected one by one, and the potential targets corresponding to the drugs were obtained. “Ulcerative colitis” was used to search for ulcerative colitis genes in the Gene Cards database. After processing and de-weighting the collected disease targets, the ulcerative colitis targets will be obtained.

#### 2.4.3 Analysis of network construction of *Saposhnikovia divaricata* in treating ulcerative colitis

The drug-target and disease-target were mapped by R language related Venny package, and the Venny diagram was drawn to get the ulcerative colitis targets of *S. divaricata*. Cytoscape 3.8.0 software was used to construct the visualized network of “Drug-active component-target-disease”.

## 3 Results

### 3.1 Results of content determination of chromogenin in root and leaf of *Saposhnikovia divaricata*

As shown in **Figure 1**, there are significant differences in chromones components in the roots and leaves of *S. divaricata*. The content of prim-*O*-glucosylcimifugin in the root of *S. divaricata* was significantly higher than that in the leaves, about 2.5 times as much as that in the leaves. However, the content of 4’-*O*-β-D-glucosyl-5-*O*-methylvisamminol in leaves was significantly higher than that in roots, about 3 times as much as that in roots. There was no significant difference in the content of cimifugin between the two groups. Sec-*O*-glucosylhamaudol was found in the roots, but not detected in the leaves. Based on these results, we found that the contents of chromogenin in the roots and leaves of *S. divaricata* were significantly different and further speculated that there must be a relationship between the transformation and accumulation of effective components. Therefore, transcriptome sequencing was used to further explore the molecular mechanism of their differences.

### 3.2 Transcriptome sequencing

#### 3.2.1 Transcriptome sequencing and quality evaluation of root and leaf of *Saposhnikovia divaricata*

Six cDNA libraries of root and leaf samples of *S. divaricata* were sequenced by Illumina NovaseqTM 6000 platform. The results were as shown in **Supplementary Table S3**. A total of 292687874 raw reads were generated, a total of 286567730 clean reads were generated after removing linkers, repeated reads and low quality reads, with Q20 > 98%, Q30 > 93%, and GC content ranging from 43.59% to 43.92%.

**Figure 2:**
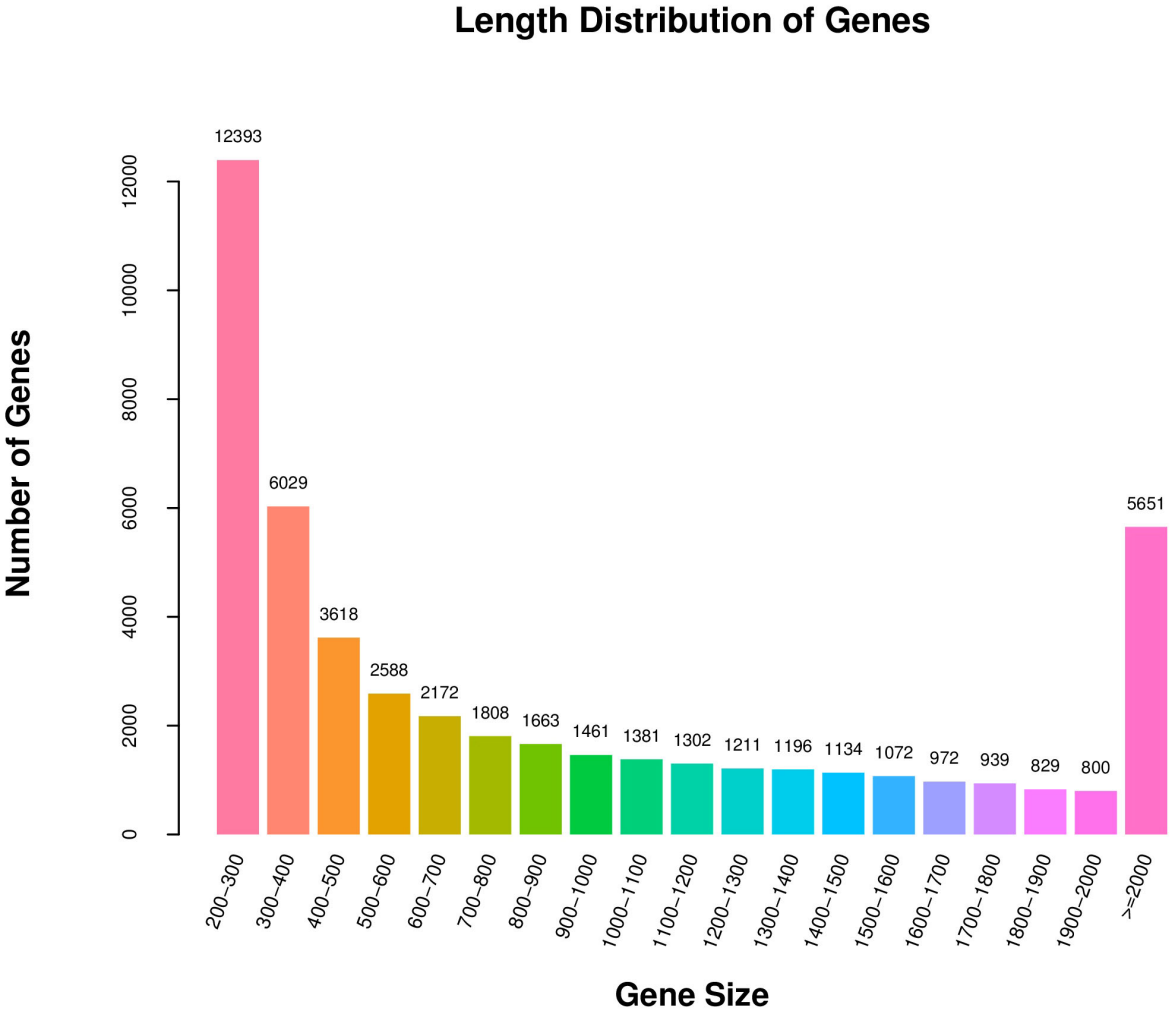
The length distribution of Unigenes in transcriptome.

Because the reference genome of *S. divaricata* was not well aligned, the comparative analysis of the non-reference transcriptome was used in this study. Trinity software was used to *de novo* assemble all clean reads from the six libraries into contigs and reflect reads back to contigs, removing redundancy, then defining the longest transcript as Unigene. Finally, 48219 Unigenes were obtained, and the N50 length was 1585 nt. The sequencing and assembly results are shown in **Supplementary Table S4**, and the length distribution of all unigenes is shown in **Figure 2**. The number of unigenes with length between 200 and 300 nt was the largest, and the number was decreasing as the length of the Unigene increased, except for Unigenes with length greater than 2000 nt.

#### 3.2.2 Unigene functional annotation analysis

In order to understand the function information of the assembled Unigenes, Diamond Software was used to perform function annotation. The database of comparison included NR, GO, Kegg, Pfam, Swiss-Prot, Egg Nog. The result of function annotation was shown in **Table 1**. All 48,219 Unigenes were annotated into the database, with 100% of them being annotated. Among them, 25,600 Unigenes were compared to GO database, accounting for 53.09%. There were 19954 Unigenes in KEGG database, accounting for 41.38%. There were 23106 Unigenes in Pfam database, accounting for 47.92%. There were 21,079 Unigenes in the Swiss-prot database, accounting for 43.72%. There were 28222 Unigenes in the Egg Nog database, accounting for 58.53%. There were 30856 Unigenes in NR database, accounting for 63.99%. We can find that the number of Unigenes compared to the NR database is the most, and the number of Unigenes compared to the KEGG database is the least.

**Table 1:** The result of a functional annotation.

DB	Num	Ratio(%)
All	48219	100.00
GO	25600	53.09
KEGG	19954	41.38
Pfam	23106	47.92
swissprot	21079	43.72
eggNOG	28222	58.53
NR	30856	63.99

**Figure 3:**
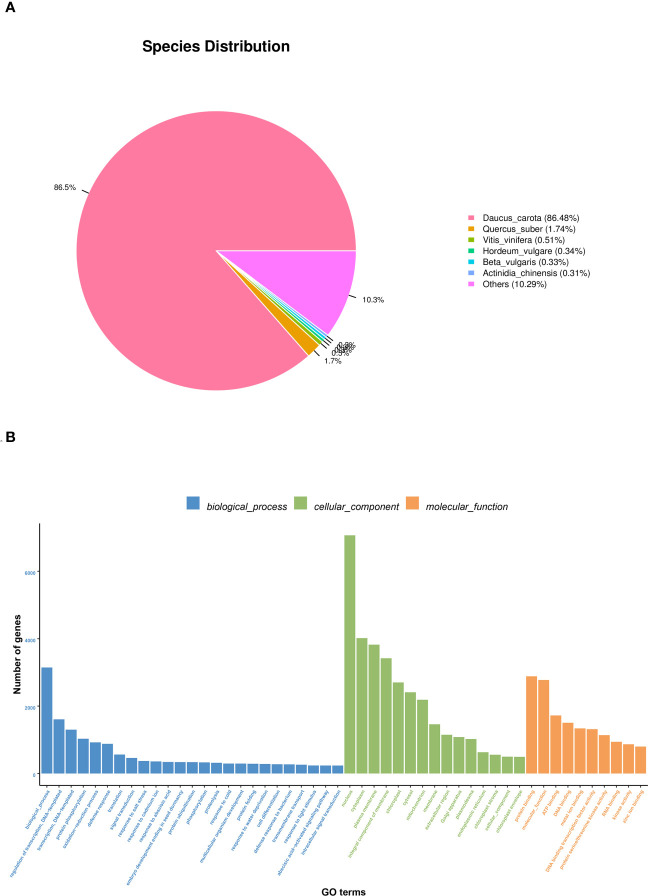
Distribution of NR homologous species **(A)** and the result of GO function annotation **(B)**.

The NR annotation results are shown in **Figure 3A**. 30856 Unigenes were annotated into the NR database. The most common species was Daucus carota (86.48%), 1.74% was homologous to Quercus suber, 0.54% to Vitis vinifera, and 0.34% to Hordeum vulgare, 0.33% and Beta vulgaris, 0.31% and Actinidia chinensis, 10.29% and other species homology. According to the results of this study, the root and leaf of *S. divaricata* have the highest homology with Daucus carota.

Go Function annotation classification information is shown in **Figure 3B**. A total of 25,600 Unigenes are annotated into 50 GO entries, classified according to Biological Process, Cellar Component, and Molecular Function. Biological Process is the most annotated Unigenes in this study, with a total of 25 entries, of which the most annotated Unigenes are Biological Process, followed by regulation of transcription regulation, DNA template andtranscription, DNA template. Next was the cell component, which was annotated to 15 entries, with the highest number of Unigenes annotated to nucleus, followed by plasma membrane and cytoplasm. The last part is molecular function. This part is annotated into 10 items, of which the number of Unigenes annotated to protein binding is the largest, followed by molecular function and ATP binding.

**Figure 4:**
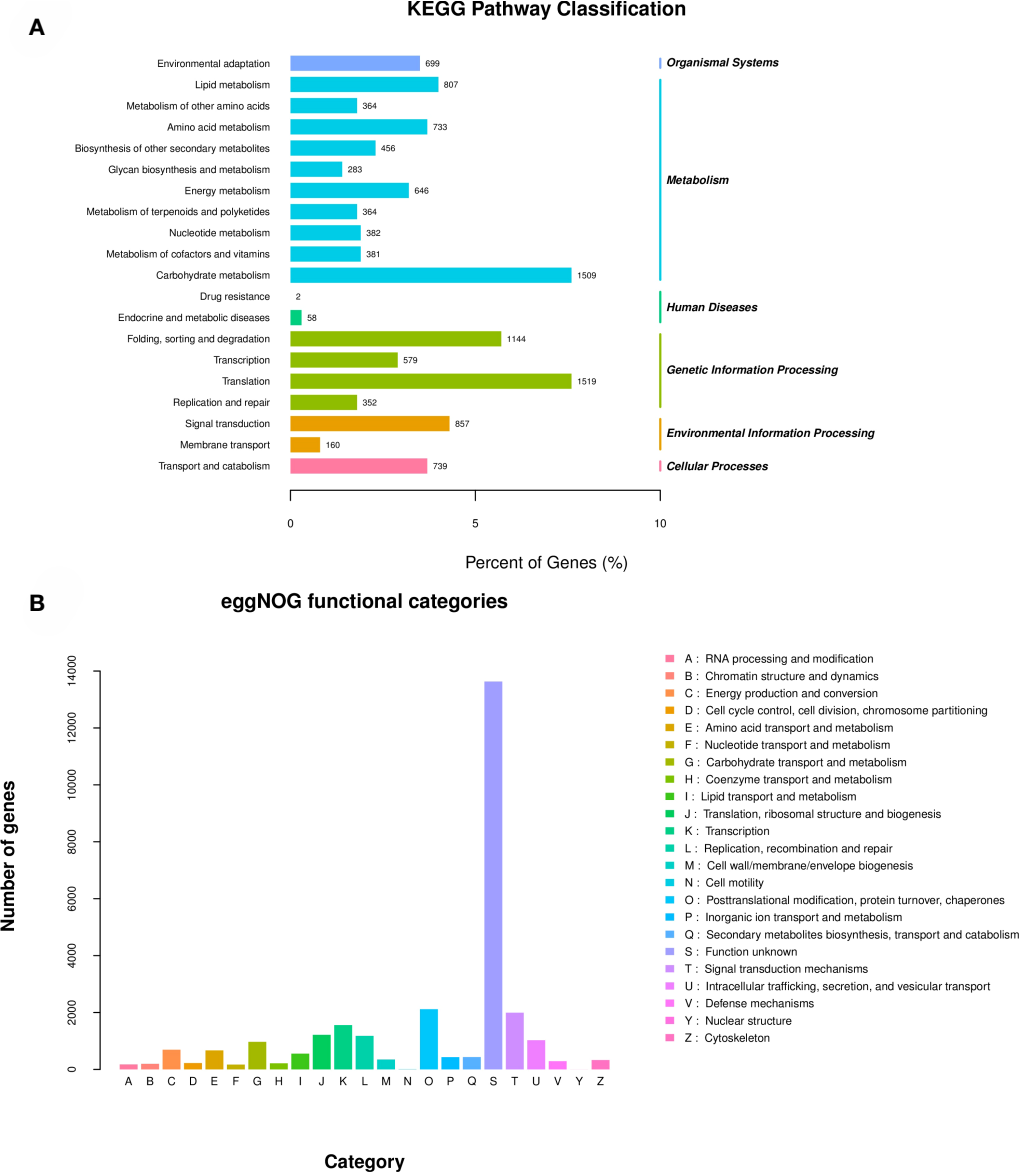
Results of the eggNOG function annotation **(A)** and the KEGG function annotation **(B)**.

The results of KEGG functional annotation to metabolic pathways are shown in **Figure 4A**. A total of 19,954 Unigenes were annotated into the KEGG database. These Unigenes were assigned to 138 metabolic pathways and were classified according to Organic Systems, Metabolism, Human Diseases, Genetic Information Processing, Environmental Information Processing and Cellular Processes (cellular process) these 6 directions are classified. Metabolism has the most annotated genes, including Carbohydrate Metabolism (1,509 genes), Lipid Metabolism (807 genes) and Amino acid Metabolism (733 genes), these are the top 3 metabolic pathways in the Metabolism direction.

The results of the eggNOG functional annotation classification are shown in **Figure 4B**, with a total of 28,222 unigenes annotated into 23 categories in this database. Among them, 13647 Unigenes were annotated to function unknown, 2118 Unigenes were annotated to Posttranslational modification, protein turnover, chaperones, 1995 Unigenes were annotated to Signal transduction mechanisms, 1559 Unigenes were annotated to Transcription, and 1221 Unigenes were annotated to Signal transduction mechanisms. For Translation, ribosomal structure and biogenesis, 1189 Unigenes were annotated to Replication, recombination and repair, and 1027 Unigenes were annotated to Intracellular trafficking, secretion, and vesicular transport. Above are the top 7 egg NOG categories with the number of annotated Unigenes.

#### 3.2.3 Functional analysis and functional annotation of differentially expressed genes in roots and leaves of *Saposhnikovia divaricata*

**Figure 5:**
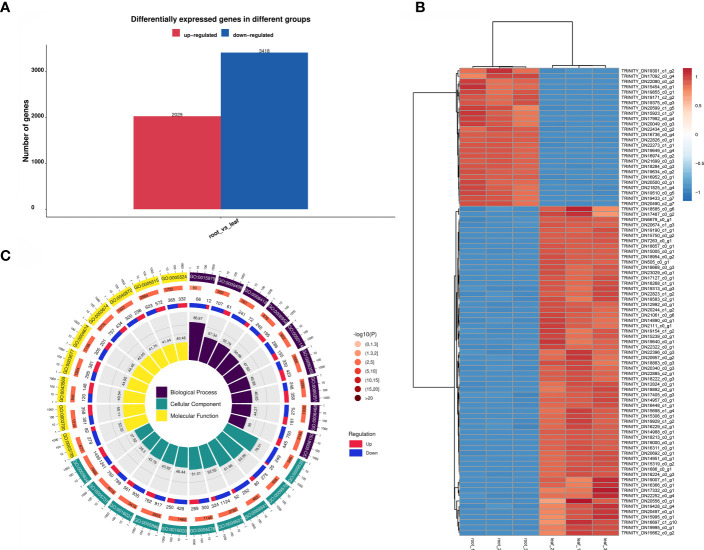
Functional analysis of differentially expressed genes. **(A)**: DEGs in the roots and leaves of *S. divaricata. ***(B)**: Cluster heatmap of differentially expressed genes in root and leaf of *S. divaricata. ***(C)**: Go mapping of differentially expressed genes in root and leaf of *S. divaricata*.

A total of 5447 DEGs were screened in the root vs. leaf comparison group, of which 2029 DEGs were upregulated and 3418 DEGs were downregulated. The number of down-regulated genes was higher than that of up-regulated genes in the root vs. leaf comparison group, which indicated that the differential genes were low-expressed in root and high-expressed in leaf. (**Figure 5A**). Cluster analysis showed that the number of down-regulated genes was significantly higher than that of up-regulated genes (**Figure 5B**). The significant enrichment result of the DEGs mapping to the secondary entry in the GO database is shown in **Figure 5C**. In Biological Process, DEGs are enriched for biological Process, regulation of transcription, DNA-templated and transcription, DNA-templated. In the Cellar Component, DEGs are enriched in terms nucleus, plasma membrane, and cytoplasm. Among the Molecular functions, the term with higher concentration of DEGs are protein binding, Molecular Function and ATP binding.

**Figure 6:**
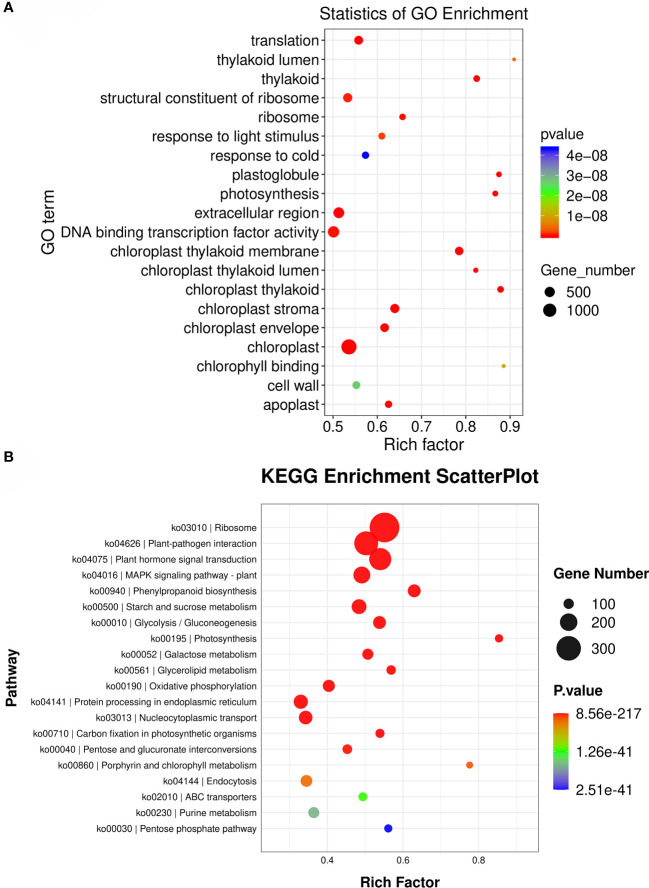
Functional annotations of differentially expressed genes. **(A)**: Enrichment analysis of air bubble diagram of GO gene differentially expressed in root and leaf of *S. divaricata. ***(B)**: Enrichment and analysis of bubble diagram of differentially expressed gene KEGG in root and leaf of *S. divaricata.*.

When enriched with lower-level GO entries, the results showed that the entries with the highest number of DEGs were chloroplast, extracellular region, and DNA binding transcription factor activity (**Figure 6A**). The enrichment of DEGs on Pathway in KEGG database was analyzed by the same method as GO enrichment analysis. The results showed that the most distributed DEGs were Ribosome, Plant-pathogen interaction, Plant hormone signal transduction and Phenylpropanoid biosynthesis (**Figure 6B**).

#### 3.2.4 Screening of key differentially expressed genes in related biosynthetic pathways

As shown in **Table 2**, 10 differentially expressed genes were screened in plant hormone signal transduction, phenylpropanoid biosynthesis and flavonoid biosynthesis. IAA, AUX, ARF were screened out in plant hormone signal transduction, 4CL and C4H were screened out in phenylpropanoid biosynthesis. CHS, F3H, CYP98A, HCT and CCOAOMT were screened from flavonoid biosynthesis. C4H and CYP98A were up-regulated in the root, while F3H was down-regulated in the root. IAA, AUX, ARF, 4CL, CHS, HCT and CCOAOMT had both up-regulated and down-regulated unigenes in the root.

**Table 2:** Analysis of differentially expressed genes in plant hormone signal transduction, phenylpropanoid and flavonoid biosynthetic pathways.

Name	Gene ID	EC ID	Root VS Leaf up/down
CHS	TRINITY_DN18872_c0_g1	EC:2.3.1.74	down
	TRINITY_DN16556_c0_g2		down
4CL	TRINITY_DN21607_c0_g4	EC:6.2.1.-	down
C4H	TRINITY_DN18261_c1_g1	EC:1.14.13.11	up
CYP98A1	TRINITY_DN6854_c0_g1		up
HCT	TRINITY_DN19267_c0_g2	EC:2.3.1.133	down
	TRINITY_DN15301_c0_g1		up
	TRINITY_DN22200_c0_g5		up
	TRINITY_DN21238_c0_g2		up
	TRINITY_DN18362_c1_g2		down
	TRINITY_DN14445_c0_g1		down
	TRINITY_DN2046_c0_g1		down
F3H	TRINITY_DN16210_c0_g1	EC:1.14.11.9	down
CCoAOMT	TRINITY_DN1651_c0_g1	EC:2.1.1.104	down
	TRINITY_DN22381_c1_g3		up
	TRINITY_DN19466_c0_g3		up
IAA4	TRINITY_DN16976_c1_g2		down
IAA16	TRINITY_DN16894_c0_g12		down
IAA14	TRINITY_DN18191_c0_g3		down
IAA4	TRINITY_DN15660_c0_g6		down
IAA4/5	TRINITY_DN17506_c0_g1		down
IAA26	TRINITY_DN19367_c0_g4		up
IAA9	TRINITY_DN17535_c0_g5		up
AUX22	TRINITY_DN15428_c0_g1		down
AUX1	TRINITY_DN21291_c0_g8		up
ARF	TRINITY_DN17520_c0_g3		down
ARF1	TRINITY_DN15870_c3_g2		down
ARF5	TRINITY_DN22318_c1_g1		up
ARF3	TRINITY_DN20451_c0_g1		up

#### 3.2.5 RT-qPCR analysis

**Figure 7:**
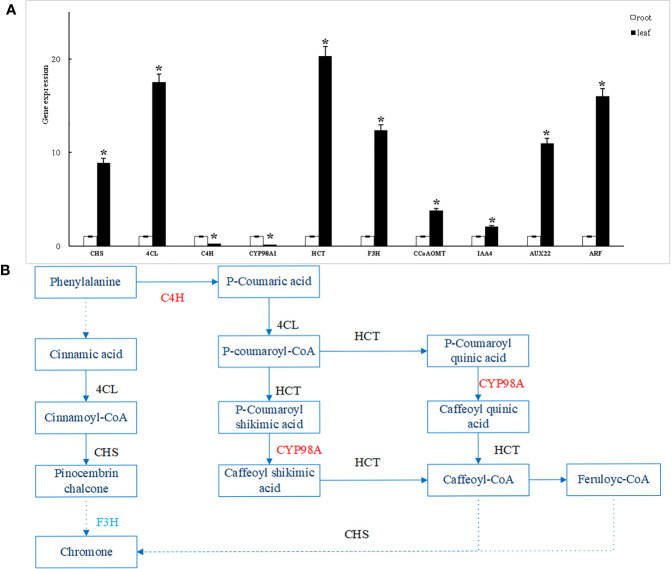
RT-qPCR validation of differentially expressed genes **(A)** and biosynthesis pathway of flavonoids in *S. divaricata ***(B)**. *: Indicates significant difference (P<0.05).

Note: In the root vs. leaf comparison group, solid Arrow indicates direct effect, dotted arrow indicates indirect effect, red font represents gene up-regulation, blue font represents gene down-regulation, black font represents gene both up-regulation and down-regulation.

To verify the reliability of the transcriptome data, RT-qPCR validation was performed on 10 randomly selected differentially expressed genes and found that the expression trends of all genes were consistent with the transcriptome data. The results demonstrate the reliability of the transcriptome data obtained in this study (**Figure 7A**).

#### 3.2.6 Prediction of flavonoid biosynthesis pathway in *Saposhnikovia divaricata*

In conclusion, the difference in the content of chromogen ketones in *S. divaricata* is the main reason for the difference in the quality of *S. divaricata*. The difference of synthesis rate and accumulation amount of chromogenic ketones in root and leaf of *S. divaricata* may be related to up-and down-regulation of related genes. Therefore, based on the screening and regulation of differential genes, the biosynthetic pathway of flavonoids in *S. divaricata* and the biosynthetic pathway of these important chromogens were predicted as follows. As shown in **Figure 7B**. The precursor of flavonoid biosynthetic pathway is phenylalanine in phenylpropane biosynthetic pathway, which produces cinnamic acid under the action of PAL (Chen et al., 2021). Then, there are two paths. One route is that cinnamic acid generates Cinnamoyl-CoA under the action of 4CL, Cinnamoyl-CoA generates Pinocembrin Chalcone under the regulation of CHS, and then produces chromogenin indirectly through the down-regulation of F3H; The other pathway is that cinnamic acid generates p-Cinnamoyl-CoA with the up-regulation of C4H and the action of 4CL, and then p-Cinnamoyl-CoA generates caffeoyl-CoA with the up-regulation of CYP98A and the Action of HCT, the chromogenic ketone is then generated under the indirect action of CCoAOMT and CHS.

### 3.3 Network pharmacology explores whether the leaves of *Saposhnikovia divaricata* can be developed and utilized

The content of chromogenin in root and leaf of *S. divaricata* was significantly different by HPLC. So we screened the key candidate genes for root and leaf content differences by transcriptomics and predicted the pathway of chromogenin biosynthesis in *S. divaricata*. In addition, the most interesting thing is that the content of 4’-*O*-β-D-glucosyl-5-*O*-methylvisamminol in the leaves of *S. divaricata* is significantly higher than that in the roots and is about three times higher than that in the roots. Such high levels of 4’-*O*-β-D-glucosyl-5-*O*-methylvisamminol are present in the leaves of *S. divaricata*, which makes us wonder if the non-medicinal parts of the leaves of *S. divaricata* may also have medicinal potential. Since network pharmacology can predict the mechanism of action and evaluate the efficacy of drugs through network relationships, we take the therapeutic effect of *S. divaricata* on ulcerative colitis as an example, the visualized network diagram of”Drug-active component-target-disease”was constructed.

#### 3.3.1 Screening of main active components in *Saposhnikovia divaricata*

After collection and arrangement, a total of 173 chemical components were obtained. Eighteen active components of *S. divaricata* were screened according to oral bioavailability (OB)≥30% and drug-like activity (DL)≥0.18. Prim-*O*-glucosylcimifugin, 4’-*O*-β-D-glucosyl-5-*O*-methylvisamminol, cimifugin, and sec-*O*-glucosylhamaudol are widely considered as active compounds in *S. divaricata* due to their high content and strong pharmacological activities. Although their oral bioavailability is less than 30%, they are considered as bioactive compounds and can be used for further analysis (Jia et al., 2016; Kim et al., 2017; Zhou et al., 2017; Fu et al., 2020; Li et al., 2020). Thus, a total of 22 compounds were obtained, as shown in **Table 3**.

**Table 3:** Bioactive compounds in *S. divaricate.*.

Mol ID	Molecule Name	MW	OB (>30%)	DL(>0.18)
MOL000011	(2R,3R)-3-(4-hydroxy-3-methoxy-phenyl)-5-methoxy-2-methylol-2,3-dihydropyrano[5,6-h][1,4]benzodioxin-9-one	386.38	68.83	0.66
MOL011730	11-hydroxy-sec-o-beta-d-glucosylhamaudol_qt	292.31	50.24	0.27
MOL011732	anomalin	426.5	59.65	0.66
MOL011737	divaricatacid	320.32	87	0.32
MOL011740	divaricatol	334.35	31.65	0.38
MOL001941	Ammidin	270.3	34.55	0.22
MOL011747	ledebouriellol	374.42	32.05	0.51
MOL011749	phelloptorin	300.33	43.39	0.28
MOL011753	5-O-Methylvisamminol	290.34	37.99	0.25
MOL002644	Phellopterin	300.33	40.19	0.28
MOL000359	sitosterol	414.79	36.91	0.75
MOL000173	wogonin	284.28	30.68	0.23
MOL000358	beta-sitosterol	414.79	36.91	0.75
MOL001494	Mandenol	308.56	42	0.19
MOL001942	isoimperatorin	270.3	45.46	0.23
MOL003588	Prangenidin	270.3	36.31	0.22
MOL007514	methyl icosa-11,14-dienoate	322.59	39.67	0.23
MOL013077	Decursin	328.39	39.27	0.38
MOL011734	Cimifugin	306.34	13.49	0.29
MOL000373	(2S)-4-methoxy-7-methyl-2-[1-methyl-1-[(2S,3R,4S,5S,6R)-3,4,5-trihydroxy-6-methylol-tetrahydropyran-2-yl]oxy-ethyl]-2,3-dihydrofuro[3,2-g]chromen-5-one	452.5	5.38	0.81
MOL011750	prim-o-beta-d-glucosylcimifugin	486.52	27	0.79
MOL011752	sec-o-beta-d-glucosylhamaudol	456.49	15.08	0.75

#### 3.3.2 Acquisition of drug targets and disease targets

**Figure 8:**
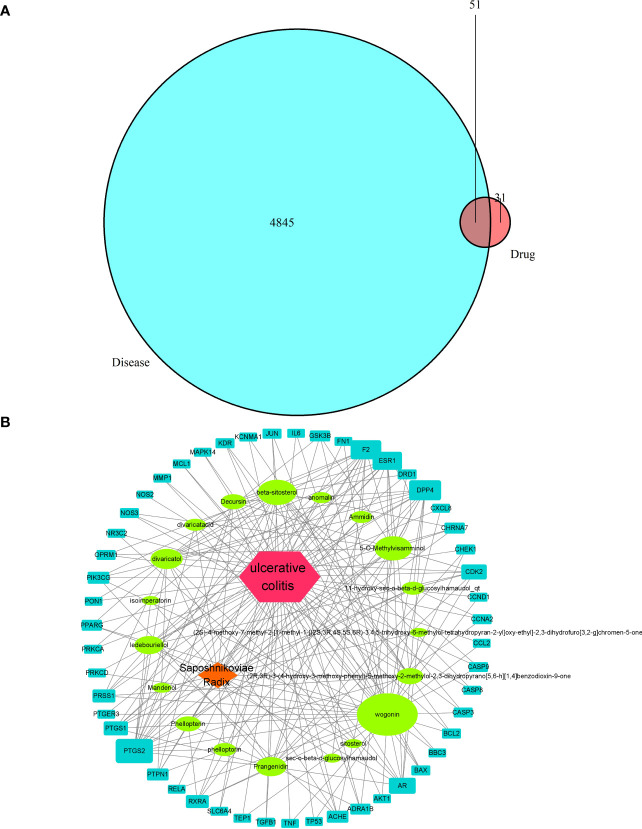
Drug-disease target intersection in Venn diagram **(A)** and visual analysis of *S. divaricata* in the treatment of ulcerative colitis **(B)**.

A total of 22 active chemical constituents of *S. divaricata* were screened from the TCMSP database (**Table 3**), and 82 drug targets were obtained after deweighting. A total of 4845 disease targets were obtained from Gene Cards database. The 82 active ingredient targets of *S. divaricata* were intersected with 4845 ulcerative colitis related genes, resulting in a total of 51 active ingredient-disease cross-targets, as shown in **Figure 8A**.

#### 3.3.3 Topology analysis of drug-component-target network and PPI network

The drug-component-target network was obtained by importing the information of drug, component and overlapping genes into cytoscape 3.8.0 software. Network analyzer is used to analyze the topology of the network, and the nodes are arranged in descending order according to the degree of nodes. Among them, the green node represents the active ingredient of wind, and the blue node represents the target of drug action, as shown in **Figure 8B**. According to **Figure 8**, Wogonin, 5-O-Methylvisamminol, divaricatol, and ledebouriellol were the most frequently cited components, most of them are chromogenic ketones. It proved that the key active ingredient of *S. divaricata* for ulcerative colitis is chromogenin, which is consistent with the results reported in the literature.

## 4 Discussion

*S. divaricata* is a major medicinal material in China, which is widely used in clinic. Chromogen ketones are the main chemical components of *S. divaricata*, and are the main active components in its antipyretic, analgesic and anti-inflammatory effects. Modern pharmacological studies show that 4’-*O*-β-D-glucosyl-5-*O*-methylvisamminol in *S. divaricata* has obvious antipyretic, analgesic, anti-inflammatory and anti-platelet aggregation effects (Zhou et al., 2017; Fu et al., 2020). At present, the supply of *S. divaricata* on the market exceeds the demand, but the phenomenon of using only the roots of *S. divaricata* and discarding the non-medicinal parts has caused a great waste of resources. It was found that the content of 4’-*O*-β-D-glucosyl-5-*O*-methylvisamminol was not lower than that of root, which proved that the non-medicinal part of *S. divaricata* might be exploited.

Plant hormones play an important role in all stages of plant growth and environmental response (Balcerowicz et al., 2021). Aux/IAA, a member of the early auxin-responsive gene family, was originally isolated from soybean. They participate in the formation of plant tissues and organs, and play an important regulatory role in plant response to stress (Luo et al., 2018; Jiang et al., 2021). ARF is an auxin-responsive factor that is regulated by hormone-sensing pathways (Peng et al., 2020). ARF and Aux/IAA are important factors in auxin signal transduction pathway, which exist widely in plants and play important regulatory roles in some physiological processes. For example, in plant hormone signaling pathways, IAA/AUX can inhibit the activity of transcription factor ARF when auxin concentrations are low, and the addition of auxin can promote the degradation of IAA/AUX proteins, activating ARF-regulated gene expression (Piya et al., 2014; Pierre-Jerome et al., 2016). The Aux/IAA family plays an important role in plant growth and development, including root, stem, leaf, flower, organ and fruit formation. It was found that the ARF-AUX/IAA-COREP interaction pattern was common. The ARF-Aux/IAA interaction could regulate not only the formation of lateral roots, but also fruit development. After screening of key genes, we infer that the root-leaf relationship of *S. divaricata* must be influenced and regulated by IAA, AUX and ARF, this discovery provides a new idea for understanding the biological significance of morphogenesis and development of different plant tissues and organs, which is worthy of further investigation.

Modern pharmacological studies show that chromogen ketone is the most important chemical component of *S. divaricata*, and chromogen ketones are mainly synthesized by phenylpropane biosynthesis pathway and flavonoid biosynthesis pathway. The biosynthetic pathway of flavonoids in Saposhnikovia divaricata and the biosynthetic pathway of these important chromogens were predicted as **Figure 7B**. Among them, 4CL and C4H are key enzymes in phenylpropanoid metabolic pathway in flavonoid biosynthesis. Some studies have shown that when the expression levels of 4CL and C4H are high in pear and other plants, the content of flavonoid also increases (Bai et al., 2021; Shahivand et al., 2021). But in Lonicera macranthoides, 4CL and C4H negatively regulate the production of flavonoids downstream (Pan et al., 2021). CYP98A belongs to cytochrome P450 (CYP450), which is involved in the biosynthesis of a large number of plant secondary metabolites, such as alkaloids, flavonoids, coumarins and terpenoids (Qi et al., 2017). CHS (chalcone synthase) and flavone-3-hydroxylase (F3H) are key enzymes in the flavonoid pathway that co-catalyze the early synthesis of flavonoid. Caffeoyl-coenzyme A-O-methyltransferase (CCOAOMT) is a class of s-adenosine-l-methionine (SAM) methyltransferase, which catalyzes the formation of methylated phenolic metabolites from flavonoids, alkaloids and other compounds (Nakatsuka et al., 2008; Qi et al., 2017; Yang et al., 2019). Comparing the root and leaf of *S. divaricata*, we found that the expression of C4H and CYP98A were higher in the root than in the leaf, and the expression of F3H was higher in the leaf than in the root. However, in previous experiments, we found that the content of 4’-*O*-β-D-glucosyl-5-*O*-methylvisamminol in leaves was significantly higher than that in roots, and the content of prim-*O*-glucosylcimifugin in roots was significantly higher than that in leaves. Therefore, we speculate that the quality difference between root and leaf of *S. divaricata* may be regulated by up-and down-regulated expression of these genes. Among them, F3H may be the key gene affecting the production of 4’-*O*-β-D-glucosyl-5-*O*-methylvisamminol, while C4H and CYP98A are the key genes regulating other chromogenic ketones in the root of *S. divaricata*.

Glycosides are one of the main active components of many traditional Chinese medicines, and have many important pharmacological activities, which are paid more and more attention by researchers. However, natural glycosides are not the best molecular structure because of their glycosyl, high polarity, low fat solubility and difficult absorption through intestinal mucosa. It needs to be metabolized into aglycones, low glycosides or other products by intestinal flora, which can make the molecular polarity decrease and the lipid solubility increase, so that it can penetrate the intestinal wall and enter the blood circulation better, and then exert the effect of medicine(Liu et al., 2003; Zhang et al., 2013). For example, Paeoniflorin has anti-inflammatory, analgesic, immunomodulatory and antigenic liver cancer effects, but can not be effectively absorbed after direct oral administration, it needs to be transformed into aglycones in the gut by the intestinal flora in order to better penetrate the intestinal wall into the blood to play a role (Ke et al., 2016). 4’-*O*-β-D-glucosyl-5-*O*-methylvisamminol also belongs to glycoside, after transformation, it can be converted into 5-O-Methylvisamminol which is easy to be absorbed by human body, and exert drug effect in human body. The content of 4’-*O*-β-D-glucosyl-5-*O*-methylvisamminol in the leaves and roots of *S. divaricata* was significantly higher than that in the roots and about 3 times higher than that in the roots. We then used the ulcerative colitis as an example to predict the function of active components of Saposhnikovia *via* network pharmacology and found that 4’-*O*-β-D-glucosyl-5-*O*-methylvisamminol plays an extremely important role. Therefore, 4’-*O*-β-D-glucosyl-5-*O*-methylvisamminol from leaves of Dioscorea rubescens is of great value for development and utilization, as long as it is converted into 4’-*O*-β-D-glucosyl-5-*O*-methylvisamminol *in vivo* or *in vitro* by certain methods, can be applied to the clinical absorption by the human body. This proves that the leaves of *S. divaricata* have high medicinal value and should not be abandoned, and can be used in the development of new drugs and clinical applications. This result provides a new idea for the exploitation and utilization of the medicinal resources of *S. divaricata*.

## 5 Conclusion

In this study, it was found that the content of chromogenin in the roots and leaves of *S. divaricata* was significantly different. The content of 4’-*O*-β-D-glucosyl-5-*O*-methylvisamminol in leaves was about three times as much as that in roots, and the content of prim-*O*-glucosylcimifugin in roots was about 2.5 times as much as that in leaves. Sec-*O*-glucosylhamaudol was found only in the root, but not in the leaf. Ten Key differential candidate genes were screened, and F3H was speculated to be the key gene affecting the production of 4’-*O*-β-D-glucosyl-5-*O*-methylvisamminol in leaves, C4H and CYP98A are the key genes regulating the other chromogenic ketones in the root of *S. divaricata*. They can be used as important candidate genes for the mechanism of quality difference of *S. divaricata*.In addition, network pharmacology demonstrated that 5-O-methylvesamitol plays an important role in the treatment of ulcerative colitis. The content of 4’-*O*-β-D-glucosyl-5-*O*-methylvisamminol in the leaves of *S. divaricata* is significantly higher than that in the roots. If the 4’-*O*-β-D-glucosyl-5-*O*-methylvisamminol can be converted into 5-O-methylvesamitol which is easy to be absorbed by the human body, the leaves of *S. divaricata* will be of high development and utilization value. This work will be useful for further research on flavonoid biosynthesis in *S. divaricata* associated molecular regulation and the development of traditional Chinese medicine.

## Data availability statement

The datasets presented in this study can be found in online repositories. The names of the repository/repositories and accession number(s) can be found below: https://www.ncbi.nlm.nih.gov/bioproject/PRJNA876052.

## Author contributions

YC, TZ and CC conceived and designed the experiments. YC and TZ performed most of the experiments and completed the first draft. YC, TZ, CC, ZX and CL conducted the experiments and carried out the analysis. All authors contributed to the article and approved the submitted version.
