# Supplementary material for: Transcriptomics explores the potential of flavonoid in non-medicinal parts of Saposhnikovia divaricata (Turcz.) Schischk
Source: Front Plant Sci. 2023 Feb 27;14:1067920. doi: 10.3389/fpls.2023.1067920 (PMC10010146; doi:10.3389/fpls.2023.1067920)
Supplement: Supplementary file 1 [file DataSheet_1.docx]

Supplementary Table S1: Standard curves of four kinds of reference components.

| Compounds | Regression equations | R^2^ | Linear ranges (mg·mL^−1^) |
| --- | --- | --- | --- |
| prim-*o*-glucosylcimifugin | *y*=2729.8*x*-686.53 | 0.998 | 2~32 |
| 4’-*O*-β-D-glucosyl-5-*O*-methylvisamminol | *y*=5418*x*-1687.2 | 0.999 | 3~48 |
| cimifugin | *y*=4443.7*x*-730.38 | 0.997 | 2.3~18.4 |
| sec-*o*-glucosylhamaudol | *y*=1556.5*x*-490.21 | 0.998 | 0.7~11.2 |


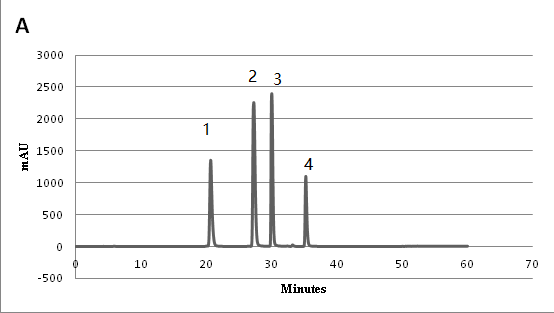


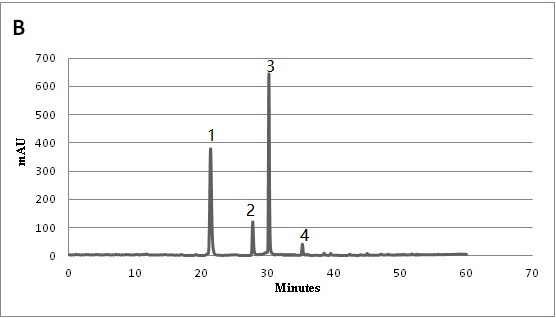


Supplementary Figure S1 HPLC chromatogram of SR reference substance solution(A) and HPLC chromatogram of test substance(B).

1: prim-o-glucosylcimifugin; 2: 4-O-β-D-glucosyl-5-O-methylvisamminol; 3: cimifugin; 4: sec-o-glucosylhamaudol

Supplementary Table S2 Primers used for real-time quantitative PCR

| Gene ID | Name | prodSize | primer (5'-3') |
| --- | --- | --- | --- |
| *GAPDH* |  | 117 | ATGGACCATCAGCAAAGGAC |
|  |  | 117 | GGTAGCACTTTCCCAACAGC |
| TRINITY_DN18872_c0_g1 | CHS | 109 | TCCTGCTAATGTGCTGGCTATTGG |
|  |  | 109 | ATCGGTCATGTGTTCGGAGTTAGTG |
| TRINITY_DN21607_c0_g4 | 4CL | 136 | CCAACTCTCCAGTACGCAACCATAC |
|  |  | 136 | AGTAAAGCACCGCAGCAGTATCG |
| TRINITY_DN18261_c1_g1 | C4H | 104 | TTGAATGGGGCATTGCGGAACTAG |
|  |  | 104 | TGGCTCACAGATTTGAACTCCAACC |
| TRINITY_DN6854_c0_g1 | CYP98A1 | 97 | GCACCCTGTGGCACCACTATTAG |
|  |  | 97 | ATTGACCAACACTACGGTTCCTTCG |
| TRINITY_DN19267_c0_g2 | HCT | 150 | CATTGCCATTCCGCCTTTCATTGAC |
|  |  | 150 | AGGACGGTTGTGGTAGGAGACTTG |
| TRINITY_DN16210_c0_g1 | F3H | 106 | GCAGGATTGGCGTGAGATAGTGAC |
|  |  | 106 | AACCTCGGTGACTGACCTCCATC |
| TRINITY_DN1651_c0_g1 | CCoAOMT | 94 | CCCTCGCATTGCCTGATGATGG |
|  |  | 94 | ACGCCAGCCTCACGAATGAATG |
| TRINITY_DN16976_c1_g2 | IAA4 | 132 | CACCACCTCCAAAGGCACAAGTAG |
|  |  | 132 | GATACGGTGCTCCATCCATGCTTAC |
| TRINITY_DN15428_c0_g1 | AUX22 | 143 | GACTGGATGCTTGTGGGAGATGTTC |
|  |  | 143 | CGACACCGTTGGTTAGCTTGAGAG |
| TRINITY_DN17520_c0_g3 | ARF | 106 | ATGAATGCTGCCGAGATGACTGAC |
|  |  | 106 | CCTCGTATAGGCCCTCTCCAGATG |

Supplementary Table S3 RNA-seq data from six samples

| Sample | Raw_Reads | Raw_Bases | Valid_Reads | Valid_Bases | Valid% | Q20% | Q30% | GC% |
| --- | --- | --- | --- | --- | --- | --- | --- | --- |
| leaf_1 | 50560316 | 7.63G | 49424718 | 6.97G | 97.75 | 98.54 | 95.36 | 43.85 |
| leaf_2 | 50682010 | 7.65G | 49339656 | 6.95G | 97.35 | 98.46 | 95.19 | 43.79 |
| leaf_3 | 49981584 | 7.55G | 48795380 | 6.87G | 97.63 | 98.47 | 95.21 | 43.63 |
| root_1 | 41486368 | 6.22G | 40820710 | 5.72G | 98.40 | 98.18 | 93.77 | 43.69 |
| root_2 | 52422016 | 7.86G | 51496090 | 7.21G | 98.23 | 98.21 | 93.91 | 43.59 |
| root_3 | 47555580 | 7.13G | 46691176 | 6.54G | 98.18 | 98.29 | 94.12 | 43.92 |

Supplementary Table S4 Transcriptome sequencing assembly results

| Index | All | GC% | Min Length | Median Length | Max Length | Total Assembled Bases | N50 |
| --- | --- | --- | --- | --- | --- | --- | --- |
| Transcript | 109293 | 40.12 | 201 | 724 | 15568 | 112108945 | 1575 |
| Gene | 48219 | 40.24 | 201 | 577 | 15568 | 45672709 | 1585 |
